# Spatial Heterogeneity in Soil Microbes Alters Outcomes of Plant Competition

**DOI:** 10.1371/journal.pone.0125788

**Published:** 2015-05-06

**Authors:** Karen C. Abbott, Justine Karst, Lori A. Biederman, Stuart R. Borrett, Alan Hastings, Vonda Walsh, James D. Bever

**Affiliations:** 1 Department of Ecology, Evolution and Organismal Biology, Iowa State University, Ames, IA, United States of America; 2 Department of Biology, Case Western Reserve University, Cleveland, OH, United States of America; 3 Department of Biological Sciences, University of Alberta, Edmonton, Alberta, Canada; 4 Department of Biology and Marine Biology, University of North Carolina–Wilmington, Wilmington, NC, United States of America; 5 Department of Environmental Science and Policy, University of California Davis, Davis, CA, United States of America; 6 Department of Applied Mathematics, Virginia Military Institute, Lexington, VA, United States of America; 7 Department of Biology, Indiana University, Bloomington, IN, United States of America; Institute for Sustainable Plant Protection, C.N.R., ITALY

## Abstract

Plant species vary greatly in their responsiveness to nutritional soil mutualists, such as mycorrhizal fungi and rhizobia, and this responsiveness is associated with a trade-off in allocation to root structures for resource uptake. As a result, the outcome of plant competition can change with the density of mutualists, with microbe-responsive plant species having high competitive ability when mutualists are abundant and non-responsive plants having high competitive ability with low densities of mutualists. When responsive plant species also allow mutualists to grow to greater densities, changes in mutualist density can generate a positive feedback, reinforcing an initial advantage to either plant type. We study a model of mutualist-mediated competition to understand outcomes of plant-plant interactions within a patchy environment. We find that a microbe-responsive plant can exclude a non-responsive plant from some initial conditions, but it must do so across the landscape including in the microbe-free areas where it is a poorer competitor. Otherwise, the non-responsive plant will persist in both mutualist-free and mutualist-rich regions. We apply our general findings to two different biological scenarios: invasion of a non-responsive plant into an established microbe-responsive native population, and successional replacement of non-responders by microbe-responsive species. We find that resistance to invasion is greatest when seed dispersal by the native plant is modest and dispersal by the invader is greater. Nonetheless, a native plant that relies on microbial mutualists for competitive dominance may be particularly vulnerable to invasion because any disturbance that temporarily reduces its density or that of the mutualist creates a window for a non-responsive invader to establish dominance. We further find that the positive feedbacks from associations with beneficial soil microbes create resistance to successional turnover. Our theoretical results constitute an important first step toward developing a general understanding of the interplay between mutualism and competition in patchy landscapes, and generate qualitative predictions that may be tested in future empirical studies.

## Introduction

Microbe-responsive plant species gain nutritional benefits from associations with soil microbial mutualists, such as mycorrhizal fungi and rhizobia, and often provision sugars to these microbes, thus promoting microbial growth. Plant species vary greatly in their responsiveness to nutritional soil microbial mutualists, however, and non-responsive plants may be neutral to the presence of soil mutualists. In general terms, legumes typically respond positively to rhizobia while non-legumes do not, and ectomycorrhizal tree species respond positively to ectomycorrhizal fungi while non-ectomycorrhizal plants do not. There is also variation among plants associating with a common mutualist, as, for example, early successional plant species often have low response to arbuscular mycorrhizal (AM) fungi, while late successional species can be highly responsive [1,2,3]. Similarly, invasive plant species in California grasslands have been found not to respond strongly to AM fungi, while native plant species have high responsiveness [4,5]. Such variation in response to nutritional mutualists is associated with a trade-off in allocation to root structures for resource uptake [6,7]. As a result, the competitive outcome between plant species can depend upon the density of soil microbial mutualists, with microbe-responsive plant species having high competitive ability in association with high densities of mutualists while plant species with low microbe response have high competitive ability with low densities of mutualists, a result repeatedly demonstrated in mesocosm manipulations [8,9,10,11,12].

Given such variation in overall responsiveness, the outcome of plant-plant interactions will depend upon local densities of mutualists, which in turn depends on the ability of the plants to support populations of the mutualists. Umbanhowar and McCann [13] demonstrated that when the most responsive plant also allows the greatest population growth of the mutualist, a positive feedback dynamic is generated and can lead to alternative stable states. However, negative feedback and local coexistence will result when the most responsive plant does not support higher population growth of the microbial mutualist. In general, we expect a positive correlation between the responsiveness of plants to particular mutualists and their investment into supporting growth of that that microbial mutualist [3], as would be the case for plant species that make associations with different microbes (e.g. legumes versus non-legumes, ectomycorrhizal hosts versus non-hosts). There are greater possibilities when considering plants that vary in their responsiveness to the same mutualists, such as AM fungi-hosting plants, but available evidence suggests that a positive correlation still exists between responsiveness of plant hosts and the degree to which they support microbial populations [5,6]. Furthermore, the positive feedback expected from such a correlation has been demonstrated [5,14].

Given that the dynamics of microbial mutualists can result in alternative stable states within a patch, initial conditions should have a strong impact on local competitive outcomes. Furthermore, if there is spatial heterogeneity in the soil microbe community, the dynamics of plants across patches will be particularly important. Soil microbial mutualists disperse independently of plants and often have much more limited dispersal than plants [3,15], which should structure the resulting dynamic. Spatial heterogeneity will also be generated by any extrinsic disturbance that locally reduces the density of the soil microbial mutualists, such as tillage [16,17].

In this paper, we use a model to examine the joint effects of temporal and spatial variation in soil mutualists and seed dispersal on the competitive dynamics of plants that vary in their responsiveness to soil mutualists. Because of the potential for alternative stable states in our general model, we interpret the model’s behavior within two distinct biological scenarios that are associated with different initial conditions: invasion and succession. We ground the description of the invasion scenario around the dynamics of California grasslands [5] where microbe-responsive native plants are superior competitors to an introduced species in the presence of a native soil microbe (AM fungi), but inferior in the absence of the microbe. When the microbe is distributed patchily, the fate of the invasion is determined by the demographic and dispersal characteristics of both plant species. We investigate how the conditions leading to establishment of the introduced species are influenced by plant dispersal between microbe-inhabited and microbe-free areas. Although the model we present is simple, and our invasion scenario is just an example that is not intended to capture all possible biological invasions, it improves our understanding of how microbe-responsive native plant communities may resist invasion in heterogeneous landscapes. It also addresses the broader question of how mutualistic communities are affected when one mutualist has limited dispersal compared with its partner.

Following the invasion study, we consider our second scenario of plant succession. Early successional plants are commonly less responsive to microbial mutualists than later successional plants [3,18]. We use our model to explore whether positive plant-soil feedbacks generated by these differences in responsiveness in a patchy landscape can explain patterns of replacement during succession, or whether additional plant and soil traits must also play a role. The use of our model in this way is particularly useful because the mechanisms underlying observed correlations between microbe responsiveness and successional stage are not yet well understood.

## Methods

### Basic model for microbe-mediated plant competition

Our model tracks the local abundances of two competing plant species. One plant (“*R*” for microbe-responsive) has a facultative association with a mutualistic soil microbe (“*M*”). The other plant (“*I*” for microbe-independent) competes with the responsive plant, but is non-responsive and thus not directly affected by the microbe. We model local competition between the two plant species using the classic Lotka-Volterra competition model, with modifications to incorporate the effect of the microbe:

$$\frac{dR}{dt}=r_{R}R\left(1-\frac{R+\gamma_{I}I}{K_{R}+\frac{M}{1+\alpha M}}\right),$$ (1A)

$$\frac{dI}{dt}=r_{I}I\left(1-\frac{I+\gamma_{R}R}{K_{I}}\right),$$ (1B)

$$\frac{dM}{dt}=\frac{\phi RM}{1+\beta M}-\delta M.$$ (1C)

The parameters *r*_*R*_ and *r*_*I*_ are the maximum plant population growth rates and *K*_*R*_ and *K*_*I*_ are the plants’ microbe-free carrying capacities. *γ*_*R*_ and *γ*_*I*_ are competition coefficients that determine how severely each species is limited by the presence of the other. The effective carrying capacity of the responsive plant increases with the local abundance of the microbe according to the saturating function $\frac{M}{1+\alpha M}$. The realized carrying capacity of the responsive plant thus ranges from *K*_*R*_ when *M* = 0 to $K_{R}+\frac{1}{\alpha }$ as *M* → *∞*. The population growth rate of the microbe increases with the local density of the responsive plant at a density dependent per capita rate, $\frac{\phi R}{1+\beta M}$. The density independent microbe death rate is *δ*.

If the microbe has very fast population dynamics relative to the plants, we can simplify the model by assuming that whenever the microbe is present in the environment, it instantaneously attains its equilibrium density on the current density of the responsive plant. That is, as long as the microbe is present,

$$M=\frac{\phi R-\delta }{\beta \delta }.$$ (2)

Otherwise, if the microbe simply is not present in the environment, *M* = 0 regardless of plant density.

We are interested in microbe-mediated competition: the situation where the identity of the dominant plant species is determined by whether or not the beneficial soil microbe is present. The first step in our analysis is to determine which parameter combinations correspond to this condition. In other words, we wish to know which parameter values allow *I* to competitively exclude *R* in the absence of the microbe, but *R* to exclude *I* in the microbe’s presence. We will use the subscript “*m*” to denote plant population densities in the presence the microbe, and the subscript “*x*” for the plant densities where the microbe is absent. When the microbe is absent, the model is the familiar Lotka-Volterra competition model,

$$\frac{dR_{x}}{dt}=r_{R}R_{x}\left(1-\frac{R_{x}+\gamma_{I}I_{x}}{K_{R}}\right),$$ (3A)

$$\frac{dI_{x}}{dt}=r_{I}I_{x}\left(1-\frac{I_{x}+\gamma_{R}R_{x}}{K_{I}}\right).$$ (3B)

To study the dynamics where the microbe is present, we substitute Eq (2) into Eq (1A) and rearrange to get,

$$\frac{dR_{m}}{dt}=r_{R}R_{m}\left(1-\frac{\left(R_{m}+\gamma_{I}I_{m}\right)\left(\delta \left(\beta -\alpha \right)+\alpha \phi R_{m}\right)}{\delta \left(K_{R}\left(\beta -\alpha \right)-1\right)+\phi \left(K_{R}\alpha +1\right)R_{m}}\right),$$ (3C)

$$\frac{dI_{m}}{dt}=r_{I}I_{m}\left(1-\frac{I_{m}+\gamma_{R}R_{m}}{K_{I}}\right).$$ (3D)

We can reduce the number of parameters necessary to describe these dynamics by employing the following substitutions: ${\tilde{R}}_{i}=\frac{R_{i}}{K_{R}}$, ${\tilde{I}}_{i}=\frac{I_{i}}{K_{I}}$, $c_{R}=\frac{\gamma_{R}K_{R}}{K_{I}}$, $c_{I}=\frac{\gamma_{I}K_{I}}{K_{R}}$, $a=\frac{\delta \left(\beta -\alpha \right)}{\phi \left(K_{R}\alpha +1\right)}$, $b=\frac{K_{R}\alpha }{K_{R}\alpha +1}$, and $k=\frac{\delta \left(K_{R}\left(\beta -\alpha \right)-1\right)}{\phi K_{R}\left(K_{R}\alpha +1\right)}$. This generates the rescaled model,

$$\frac{d{\tilde{R}}_{x}}{dt}=r_{R}{\tilde{R}}_{x}\left(1-{\tilde{R}}_{x}-c_{I}{\tilde{I}}_{x}\right)$$ (4A)

$$\frac{d{\tilde{I}}_{x}}{dt}=r_{I}{\tilde{I}}_{x}\left(1-{\tilde{I}}_{x}-c_{R}{\tilde{R}}_{x}\right)$$ (4B)

without the microbe, and

$$\frac{d{\tilde{R}}_{m}}{dt}=r_{R}{\tilde{R}}_{m}\left(1-\frac{\left({\tilde{R}}_{m}+c_{I}{\tilde{I}}_{m}\right)\left(a+b{\tilde{R}}_{m}\right)}{k+{\tilde{R}}_{m}}\right)$$ (4C)

$$\frac{d{\tilde{I}}_{m}}{dt}=r_{I}{\tilde{I}}_{m}\left(1-{\tilde{I}}_{m}-c_{R}{\tilde{R}}_{m}\right)$$ (4D)

with the microbe. We analyze these rescaled models to find the parameter values that correspond to microbe-mediated competition.

Note that all of the parameters in this model must be positive for the model to make biological sense (e.g. for the microbe to benefit the responsive plant, and for the plants’ relationship to be competitive). With positive *a* and *k*, $\frac{a}{k}\left(=\frac{K_{R}\left(\beta -\alpha \right)}{K_{R}\left(\beta -\alpha \right)-1}\right)>1$. By looking at how the rescaled parameters are defined, we can see that *b* < 1. Therefore, $\frac{a}{k}>1$ also implies $\frac{a}{k}>b$. This is meaningful, since the relationship between $\frac{a}{k}$ and *b* determines the shape of the function $\frac{a+b{\tilde{R}}_{m}}{k+{\tilde{R}}_{m}}$, which in turn determines how the strength of density-dependence in (4c) responds to increasing ${\tilde{R}}_{m}$. With $\frac{a}{k}>b$, $\frac{a+b{\tilde{R}}_{m}}{k+{\tilde{R}}_{m}}$ decreases from $\frac{a}{k}$ to *b* as ${\tilde{R}}_{m}$ increases from 0 to ∞. This means that the term $\frac{a+b{\tilde{R}}_{m}}{k+{\tilde{R}}_{m}}$ weakens the microbe-responsive plant’s density-dependence as ${\tilde{R}}_{m}$ increases. This weakening represents the beneficial effect of having more microbe present when the responsive plant is more abundant. Furthermore, $\frac{a}{k}>1$ means that at low ${\tilde{R}}_{m}$, density dependence is actually stronger than it would be if there were no microbe. This means that one feature of our model is a microbe-induced Allee effect in the responsive plant population.

### Effects of seed dispersal in a patchy landscape

**Fig 1:**
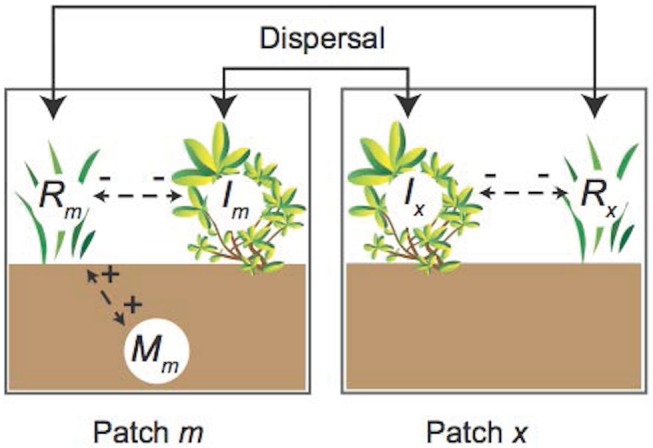
Conceptual diagram of the model. The microbe (*M*_*m*_) and microbe-responsive plant (*R*_*m*_) have a mutually beneficial relationship (+ +) in patch *m*, whereas the microbe is absent from patch *x*. In both patches, the responsive (*R*_*m*_, *R*_*x*_) and non-responsive (*I*_*m*_, *I*_*x*_) plants compete (--). Double-headed arrows represent seed dispersal between patches.

In a disturbed landscape where the microbe is patchily distributed, there will be some movement of plant seeds between areas with and without the microbe. To study the effects of this, we imagine two patches, one with (patch *m*) and one without (patch *x*) the microbe (Fig 1). We assume that the responsive plant population disperses from one spatial patch to the other at rate *D*_*R*_ and that the non-responsive plant disperses at rate *D*_*I*_. We include no microbe dispersal in our model because we assume that the patches are sufficiently far apart, or that patch *x* is sufficiently inhospitable to the microbe, that microbes from patch *m* cannot successfully colonize patch *x*. Although this assumption is likely not realistic for all soil microbes, we expect it holds for many that have the type of patchy distribution that we are interested in here. With plant dispersal, our model becomes

$$\frac{d{\tilde{R}}_{x}}{dt}=r_{R}{\tilde{R}}_{x}\left(1-{\tilde{R}}_{x}-c_{I}{\tilde{I}}_{x}\right)+D_{R}\left({\tilde{R}}_{m}-{\tilde{R}}_{x}\right)$$ (5A)

$$\frac{d{\tilde{I}}_{x}}{dt}=r_{I}{\tilde{I}}_{x}\left(1-{\tilde{I}}_{x}-c_{R}{\tilde{R}}_{x}\right)+D_{I}\left({\tilde{I}}_{m}-{\tilde{I}}_{x}\right)$$ (5B)

$$\frac{d{\tilde{R}}_{m}}{dt}=r_{R}{\tilde{R}}_{m}\left(1-\frac{\left({\tilde{R}}_{m}+c_{I}{\tilde{I}}_{m}\right)\left(a+b{\tilde{R}}_{m}\right)}{k+{\tilde{R}}_{m}}\right)+D_{R}\left({\tilde{R}}_{x}-{\tilde{R}}_{m}\right)$$ (5C)

$$\frac{d{\tilde{I}}_{m}}{dt}=r_{I}{\tilde{I}}_{m}\left(1-{\tilde{I}}_{m}-c_{R}{\tilde{R}}_{m}\right)+D_{I}\left({\tilde{I}}_{x}-{\tilde{I}}_{m}\right).$$ (5D)

### Model analysis

We use a combination of linear stability analysis and simulations to explore the behavior of this model. We begin by analyzing the limiting cases, *D*_*R*_ = *D*_*I*_ = 0 (as in Eq (4)) and *D*_*R*_ = *D*_*I*_ = ∞, to gain an understanding of how the equilibrium behavior of the model changes across the range of possible dispersal rates. The *D*_*R*_ = *D*_*I*_ = 0 case is informative in two ways: it tells us what the model’s behavior should be for arbitrarily small dispersal rates [19] and it allows us to identify which parameter values lead to which competitive outcomes in each soil type absent any seed dispersal. The *D*_*R*_ = *D*_*I*_ = ∞ case shows the model’s behavior at the other extreme. Together, these limiting cases aid interpretation of our results under the positive, finite dispersal rates (0 < *D*_*R*_, *D*_*I*_ < ∞) we expect to see in nature.

For 0 < *D*_*R*_, *D*_*I*_ < ∞, stability criteria for equilibria at which the responsive plant is absent can be derived analytically. The equilibrium where the responsive plant is present and the non-responsive plant is absent must be solved numerically, and the equilibrium at which the species coexist was found by simulation. In the latter case, we repeated our simulations from 10 sets of randomly selected initial conditions for each parameter combination considered.

The possibility for positive feedbacks and alternative stable states means that initial conditions are likely to be very important in this model. We therefore interpret the results of our stability analysis in the context of two scenarios with different initial conditions: an invasion scenario and a succession scenario. For the invasion scenario, we use California grasslands as our inspiration and imagine a microbe-responsive native plant that is initially common, confronted with a non-responsive introduced plant that is initially rare. In the succession scenario, we consider replacement of an initially common non-responsive, early successional plant by an initially rare, responsive, later successional plant.

## Results

### Basic model for microbe-mediated plant competition

**Fig 2:**
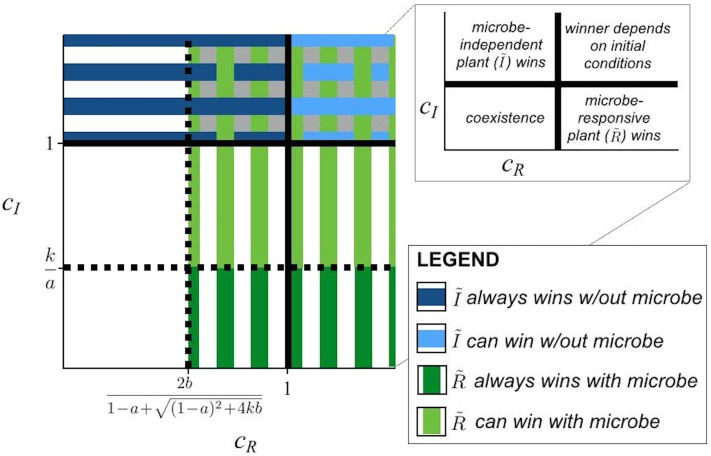
Four possible outcomes of the model. The inset shows outcomes in the absence of seed dispersal: coexistence with low *c*_*R*_ and *c*_*I*_; competitive exclusion of $\tilde{I}$ by $\tilde{R}$ with high *c*_*R*_ and low *c*_*I*_; exclusion of $\tilde{R}$ by $\tilde{I}$ with high *c*_*I*_ and low *c*_*R*_; and when both *c*_*R*_ and *c*_*I*_ are large, one plant species will exclude the other but which one depends on the initial conditions. Black solid lines delineate these 4 regions in the absence of the microbe and black dashed lines delineate the regions in the presence of the microbe. We are interested in the situation where (i) $\tilde{I}$ is dominant in the absence of the microbe, either inevitably (from all initial conditions; dark blue horizontal stripes) or at least from some initial conditions (light blue horizontal stripes), and (ii) $\tilde{R}$ is dominant in the presence of the microbe, from all (dark green vertical stripes) or some (light green vertical stripes) initial conditions. Light gray shading in the upper right shows where both (i) and (ii) hold; this is our parameter range of interest described by inequalities (6).

When there is no seed dispersal and the microbe is entirely absent (Eq (4A–4B)), our model is the familiar Lotka-Volterra competition model. Competitive exclusion of ${\tilde{R}}_{x}$ by ${\tilde{I}}_{x}$ is possible whenever *c*_*I*_ > 1; if *c*_*R*_ < 1 this result is guaranteed, but if *c*_*R*_ > 1 the outcome of competition will depend on the initial conditions. The local dynamics without seed dispersal and in the presence of the microbe are given by Eq (4C–4D). For ${\tilde{R}}_{m}$ to exclude ${\tilde{I}}_{m}$ requires, at a minimum, that $c_{R}>\frac{2b}{1-a+\sqrt{{\left(1-a\right)}^{2}+4kb}}$ (S1 Appendix). If $c_{I}<\frac{k}{a}$, ${\tilde{R}}_{m}$ will always exclude ${\tilde{I}}_{m}$, but otherwise the outcome of competition will again depend on initial conditions (Fig 2).

Recall that because we are interested in microbe-mediated competition, we wish to identify the parameter range for which ${\tilde{I}}_{x}$ can exclude ${\tilde{R}}_{x}$ *and* ${\tilde{R}}_{m}$ can exclude ${\tilde{I}}_{m}$. From the results in the preceding paragraph, we see that this situation corresponds to,

$$c_{R}>\frac{2b}{1-a+\sqrt{{\left(1-a\right)}^{2}+4kb}}$$ (6A)

$$c_{I}>1$$ (6B)

(Fig 2). In this range, the microbe-responsive plant population will exclude the non-responsive plant, if the microbe is ubiquitous, from some initial conditions. In the absence of the microbe, parameters in range (6) result in exclusion of the responsive plant by the microbe-independent plant from some or all initial conditions.

### Effects of seed dispersal in a patchy landscape

With *D*_*R*_ = *D*_*I*_ = ∞, the only stable equilibrium is exclusion of the responsive by the non-responsive plant in both patches (S1 Appendix). For arbitrarily small dispersal rates, the equilibrium behavior should be the same as the *D*_*R*_, *D*_*I*_ = 0 case [19]. As dispersal rates increase and the patches are increasingly well-mixed we expect to approach the *D*_*R*_, *D*_*I*_ = ∞ result, with total competitive exclusion of the responsive plant by the microbe-independent plant.

Between these extremes (0 < *D*_*R*_, *D*_*I*_ < ∞), we see additional model behaviors. First, we find that for any non-negligible rates of dispersal, it is impossible for the independent plant to be present in only one patch while the responsive plant is present only in the other (as is possible if *D*_*R*_, *D*_*I*_ = 0). Intuitively, this is because the competitively weaker species will be constantly reintroduced by dispersal from the other patch. The model instead has three non-trivial equilibria representing exclusion of the microbe-independent plant in both patches, exclusion of the microbe-responsive plant in both patches (as in the *D*_*R*_, *D*_*I*_ = ∞ case), or coexistence in both patches.

The equilibrium where the responsive plant is excluded from both patches is always stable under condition (6b) (S1 Appendix), in agreement with the model’s behavior under both 0 and infinite dispersal. At this equilibrium, the non-responsive plant attains its carrying capacity, ${\tilde{I}}_{m}={\tilde{I}}_{x}=1$. While this equilibrium is always stable, we do not invariably expect to observe it: if the initial conditions are far from it and there is another stable equilibrium (a situation that is possible, although not inevitable, under conditions (6); S1 Appendix), we would instead see convergence onto that other equilibrium.

In all, we found three possible outcomes with 0 < *D*_*R*_, *D*_*I*_ < ∞: (i) the non-responsive plant excludes the responsive plant everywhere on the landscape, from all initial conditions; (ii) the non-responsive plant excludes the responsive plant everywhere on the landscape from *some* initial conditions and otherwise, the responsive plant excludes the non-responder everywhere; or (iii) the non-responsive plant excludes the responsive plant from some initial conditions and otherwise the two plants coexist in both patches. For the cases with multiple stable equilibria (ii and iii), the actual behavior of the system will depend on the initial conditions. We therefore complete our model analysis in the context of our two scenarios with different initial conditions.

### Scenario 1: Invasion

The grasslands of California are one example of a largely microbe-responsive native community being invaded by microbe-independent introduced species [4,5]. Under this scenario, responsive plants are initially common and non-responders are initially rare so invasions begin near the $\tilde{R}$-only equilibrium. While exclusion of $\tilde{R}$ throughout the landscape is always a stable outcome, these initial conditions are far from it so we only expect to see deterministic extinction of the responsive native plant if this is the *only* stable outcome. When one of the other solutions is also stable, we expect that other outcome to be the one that is realized.

**Fig 3:**
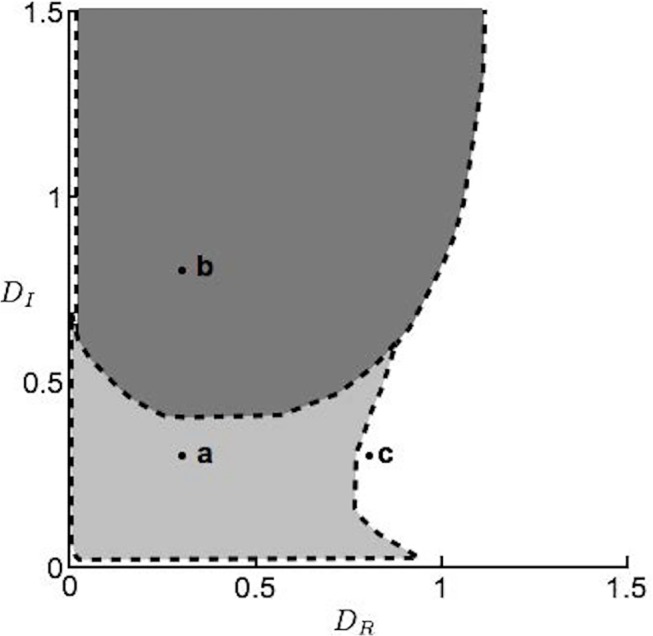
Stability regions for each of model (5)’s equilibria. The equilibrium at which the microbe-independent plant excludes the microbe-responsive plant in both patches is always stable; white regions indicate where this is the only stable equilibrium present. In the dark gray region, the equilibrium at which the responsive plant excludes the microbe-independent plant is stable and in the light gray region, the two species stably coexist. In this figure, *r*_*R*_ = 1.5, *r*_*I*_ = 1.5, *c*_*R*_ = 0.65, *c*_*I*_ = 1.1, *a* = 1.2, *b* = 0.01, and *k* = 1. See S2 Appendix for analogous figures using different parameter values. Points a, b, and c mark parameter combinations used in the corresponding panels of Fig 4.

With these initial conditions, we found that high dispersal is generally detrimental to persistence (Fig 3). With low *D*_*R*_ and *D*_*I*_, we expect stable coexistence in both patches. With increasing *D*_*I*_, the coexistence equilibrium becomes unstable and we instead expect exclusion of the introduced plant in these cases. Finally, with increased *D*_*R*_, exclusion of the native plant becomes the only stable outcome (bringing us to the outcome of the model with *D*_*R*_, *D*_*I*_ = ∞). Thus, we conclude that a slowly dispersing, microbe-responsive native plant is best equipped to resist invasion by a fast dispersing, non-responsive introduced plant in this patchy landscape.

The other parameters affect the exact ranges of dispersal rates that lead to each of these outcomes. The range of *D*_*R*_, *D*_*I*_ combinations that lead to exclusion of the introduced plant by the native plant becomes larger if we increase *r*_*R*_, *c*_*R*_, or *k*, or decrease *r*_*I*_, *a*, or *b* (S2 Appendix). Coexistence occurs for a wider range of *D*_*R*_ values if we increase *r*_*R*_, *c*_*R*_, or *k*, or decrease *c*_*I*_, *a*, or *b*. Higher *r*_*I*_, *a*, or *b*, or lower *c*_*R*_ or *k* allows coexistence over a wider range of *D*_*I*_. With any of these changes, though, the qualitative pattern of how the model’s behavior changes across dispersal rates remains as shown in Fig 3 (S2 Appendix).

**Fig 4:**
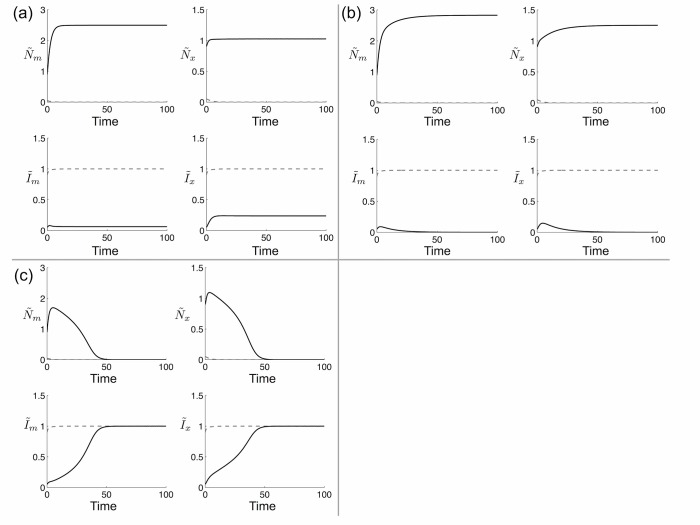
Example time series showing the possible behaviors of the model. Dashed gray lines show the dynamics from the initial conditions ${\tilde{R}}_{m},{\tilde{R}}_{x}=0.05$, ${\tilde{I}}_{m},{\tilde{I}}_{x}=0.9$ and show that exclusion of the microbe-responsive plant is always one stable outcome. Solid black lines show the additional possible behaviors, from the initial conditions ${\tilde{R}}_{m},{\tilde{R}}_{x}=0.9$, ${\tilde{I}}_{m},{\tilde{I}}_{x}=0.05$: (a) Coexistence, (b) exclusion of the microbe-independent species, and (c) exclusion of the microbe-responsive species (regardless of initial conditions). In all panels, *r*_*R*_ = 1.5, *r*_*I*_ = 1.5, *c*_*R*_ = 0.65, *c*_*I*_ = 1.1, *a* = 1.2, *b* = 0.01, and *k* = 1 as in Fig 3. The values of *D*_*R*_ and *D*_*I*_ are different for each panel and correspond to the labeled points in Fig 3: (a) *D*_*R*_ = 0.3, *D*_*I*_ = 0.3; (b) *D*_*R*_ = 0.3, *D*_*I*_ = 0.8; (c) *D*_*R*_ = 0.8, *D*_*I*_ = 0.3.

Time series of the dynamics for several different parameter combinations are shown in Fig 4. Although it is of course always possible for the introduced species to stably exclude the native species (dashed gray lines, Fig 4A–4C), it will only do so from realistic initial conditions when no other equilibria are stable (black lines, Fig 4C). When the native plant persists in both patches, its abundance is expectedly higher in patch *m* due to the beneficial effect of the microbe (Fig 4A–4B). Indeed, even in the microbe-free patch the native plant benefits from this effect: for instance, when the introduced plant is excluded, ${\tilde{R}}_{x}$ would have a density of 1 without dispersal, but with dispersal we see it is > 1 (Fig 4B). When the two species coexist, the higher density of the native plant in patch *m* also results in a lower density of the invader in that patch (Fig 4A).

### Scenario 2: Succession

Dependence on beneficial soil microbes and the quality of plants as microbial hosts both tend to increase during plant succession [3,18]. We can apply our model in a successional context by thinking of $\tilde{I}$ as a microbe-independent early successional species and $\tilde{R}$ as a microbe-dependent later successional species. The initial conditions for this scenario would thus have ${\tilde{I}}_{x}$ and ${\tilde{I}}_{m}$ near the single-species equilibrium at 1, and ${\tilde{R}}_{x}$ and ${\tilde{R}}_{m}$ near 0.

Exclusion of the responsive plant by the microbe-independent plant is always one of the stable outcomes of our model. With initial conditions at or near this solution, as we expect during early succession, the non-responsive plant will competitively exclude the responsive species with any parameter values satisfying inequalities (6) (S1 Appendix), regardless of whether there is theoretically another stable outcome. In other words, the mechanisms included in our model do not explain replacement of non-responsive plants by responsive plants during succession.

## Discussion

### Role of mutualistic soil microbes in invasion resistance

The combination of a patchy microbe distribution and two plant species experiencing microbe-mediated competition in essence creates a pair of source-sink metapopulations. For a microbe-responsive native species that relies on soil microbes like AM fungi to confer competitive dominance, microbe-rich areas act as sources with positive intrinsic population growth and microbe-free areas are sinks, in which the native plant would be competitively excluded without immigration from the source. For a microbe-independent introduced species, the opposite will be true: the microbe-free areas are sources and the microbe-rich areas are sinks. Whether or not the introduced plant can invade depends on the success of both species in both types of patches, with the native plant successfully resisting invasion only if its combination of intrinsic growth and dispersal allows it to suppress the invader throughout the landscape. Excess dispersal by either species, relative to its competitor, can lead to collapse of its source population and landscape-level extinction.

Continual dispersal of both species precludes any equilibria where either species is present in one patch but absent in the other. This means that if the introduced plant is able to establish anywhere in the landscape, the native plant will be unable to fully exclude it from even the microbe-rich areas. Thus, the spatial heterogeneity in competitive hierarchy results in spatial heterogeneity of species abundances, but not heterogeneity in the presence of species, as any species that is able to persist in its source habitat in the presence of dispersal will also persist in its sink.

The existence of multiple stable states in our model, and particularly the stability across our whole parameter range of the equilibrium at which the microbe-responsive native plant is extinct, is concerning from a management perspective. Even when the plants’ demographic and dispersal rates are such that the native plant initially persists, a disturbance that severely reduces native plant density might cause this system to switch to the stable equilibrium at which the native plant is excluded. This is especially a threat under parameter combinations that lead initially to coexistence (low *D*_*R*_ and *D*_*I*_) because this coexistence guarantees that the invader will be present at the right time to exploit any such disturbances if they arise. Examples of such disturbances include outbreaks of herbivores or pathogens that specialize on the native plant or weather conditions that are unfavorable for the native plant but not the introduced plant. A disturbance that significantly reduces microbe abundance could also have this effect. Because of these multiple stable states, microbe-mediated invasion resistance provides relatively weak protection for microbe-responsive native plants that live in temporally-variable environments.

We note that the mycorrhizal responsiveness of plant species is context dependent and can have a significant phylogenetic component [20,21], and that introduced plant species are not always less responsive or poorer hosts for AM fungi compared to native plant species with which they compete [4,22,23,24,25]. In the case where the mycorrhizal dependence of the native and non-native plants is reversed, native dominance may be facilitated by disruption of the AM fungal community. This situation—invasion of a mycorrhizal-responsive species into an established non-responsive population—parallels our succession scenario, where indeed microbe-independent species are able to maintain dominance.

### Role of beneficial soil microbes in successional transitions

The positive feedbacks in our model are sufficiently strong to prevent an established microbe-independent species from being displaced by a responsive species that depends on soil microbes for competitive dominance. Such replacements occur during succession [1,2,3] and our model’s failure to predict this is informative: it indicates that successional shifts must involve additional processes not included in our study, such as interactions with soil pathogens [3,26,27], shifts in the nature of plant-soil feedbacks over the course of succession [28], or competitive hierarchies outside of those we considered. An easy way to achieve realistic successional replacements in our model would be to look outside the parameter range described by inequalities (6). These inequalities ensure that the superior competitor can (from at least some initial conditions) change in the presence of the soil mutualist; in other words, it reflects a trade-off between competitive ability without the microbe, and competitive ability with it. If we relax this trade-off and suppose that later successional species are competitively dominant regardless of the soil biota, then of course the later successional species would invade an established population of non-responsive early successional plants. This is not very interesting, but may in fact describe some successional changes when the later successional species is simply a late arrival due to dispersal limitation or some other factor [29].

More interesting is the possibility that some early successional plants are adequate hosts for soil microbes like AM fungi, despite not responding to the presence of fungi. If this is the case, then the density of soil mutualists might build up before the arrival of the later successional responsive plant, alleviating the microbe-mediated Allee effect and allowing the responsive plant to invade. Because we modeled microbe abundance as being strictly dependent on the responsive plant’s abundance, this is not an effect we can explore in our model directly. We are unsure of whether this effect occurs in nature, but presumably there is some interspecific variation in the relationship between responsiveness to, and ability to host, soil mutualists. Perhaps successional replacements occur when good mycorrhizal hosts that happen to be relatively non-responsive are confronted by later successional responsive plants.

Finally, plants associate with soil pathogens in addition to soil mutualists and it is possible that successional changes occur when species-specific pathogens on early successional plants have accumulated to the point where those plants are no longer fit in that environment [3,26,27]. Extensions of our model that includes these later effects (variation in the relationship between the benefit derived from mutualists and the ability to host them, and soil pathogens) would likely aid understanding of the role of soil microbes in succession.

### General effect of microbial soil mutualists on plant competition

A major effect of the microbe in our model was to shrink the range of parameter values leading to plant coexistence and expand the range where initial conditions determine the winner of competition (Fig 2). Our analysis further showed that total exclusion of a microbe-responsive plant was always theoretically possible (e.g. gray dashed lines in Fig 4) but the opposite was not true: regardless of initial conditions, there are some parameter combinations for which the microbe-independent plant will never be excluded (e.g. black lines in Fig 4C). This asymmetry comes from our conditions for microbe-mediated competition (inequalities (6)), which always produce a priority effect where the microbe is present (S1 Appendix). This arises because the responsive plant’s dependence on the microbe, which is rare when its host plant is rare, creates an Allee effect. The result is that any competition coefficients that allow stable exclusion of the responsive plant in the absence of the microbe will also result in stable exclusion of a very small population of the responsive plant in the presence of the microbe. This represents a real competitive disadvantage to rare plants that rely on a density-dependent mutualist.

One assumption of our model is that the microbe’s population dynamics are very fast relative to plant dynamics. If we instead assume that population growth in all three species is occurring on the same timescale, slower accumulation of microbes should more severely limit the responsive plant’s rate of increase when rare, making it more difficult to exclude a microbe-independent species from at least some initial conditions. A complete theoretical exploration of a spatial version of model (1) would likely lead to additional interesting insights on the behavior of patchy systems with slow-growing soil microbes.

Umbanhowar and McCann [13] demonstrated that the outcome of competition between mycorrhizal plants is highly sensitive to the correlation between a plant’s response to microbes and its ability to promote microbial population growth. In our model, we considered a non-responsive plant that was truly microbe-independent, experiencing no effect from and having no effect on microbe dynamics. This decision was motivated by empirical evidence for a positive correlation between the benefit received from, and paid to, mutualist microbes [5,6]. However, the modeling framework we propose is fully adaptable to other situations; for instance, allowing the microbe to respond also, or only, to density of the non-responsive plant [30] would require a simple modification to Eq (1C). Reanalysis of the model under such a modification would likely be interesting, and informative for any situations where we expect a negative relationship between a plant’s ability to derive benefit from, and support population growth of, mutualistic microbes.

There are many examples in the literature where plant-soil feedbacks have facilitated plant invasion into new habitats [22,31,32,33]. In the model presented here, our conservative assumption was that the non-responsive plant simply tolerated the soil microbial community and did not have any direct negative effects on it. Even under this conservative assumption of no feedback between the non-responsive plant and the soil biota, the non-responsive plant is competitively dominant under a wide range of conditions. This suggests that simply maintaining beneficial plant-soil feedbacks will not always suffice to preserve a population of microbe-responsive plants. If a microbe-independent plant becomes established but the presence of the microbe-responsive species is more desirable (as in our invasion example), management may require not only removal of the microbe-independent plant, but also reintroduction of the soil mutualists [33,34,35].

The evidence that we provide of microbe-mediated alternative stable states has important implications for landscape patterns of community types. Borders between areas dominated by microbe-responsive plants and microbe-independent plants can be stabilized by the Allee effect, potentially allowing long-term persistence of alternative community types [36,37]. In fact, the California landscape appears to meet this expectation, as borders between areas dominated by native plant species and areas dominated by introduced plant species are consistent across many years in spite of abundant opportunities for dispersal and intermixing [38].

## Supporting Information

S1 Appendix Analysis of the model. (PDF)

S2 Appendix Effects of each parameter on stability of the equilibria. (PDF)
