# Supplementary material for: Spatial Heterogeneity in Soil Microbes Alters Outcomes of Plant Competition
Source: PLoS One. 2015 May 6;10(5):e0125788. doi: 10.1371/journal.pone.0125788 (PMC4422530; doi:10.1371/journal.pone.0125788)
Supplement: S1 Appendix — (PDF) [file pone.0125788.s001.pdf]

S1 Appendix: Analysis of model

Basic model for microbe-mediated plant competition: the $D_R = D_I = 0$ case

The absence of the microbe in patch x means that with no dispersal, patch x is governed by the well-known Lotka-Volterra competition model. In this model, $[\tilde{R}_x^* = 0, \tilde{I}_x^* = 1]$ is stable if $c_I > 1$. $[\tilde{R}_x^* = 1, \tilde{I}_x^* = 0]$ is stable if $c_R > 1$. Notice that both of these equilibria are stable (i.e. we have alternative stable states) when $c_R > 1$ and $c_I > 1$.

With no dispersal, patch m also has two equilibria at which one plant excludes the other: $\left[\tilde{R}_m^* = \frac{1-a+\sqrt{(1-a)^2+4kb}}{2b}, \tilde{I}_m^* = 0\right]$ and $[\tilde{R}_m^* = 0, \tilde{I}_m^* = 1]$. The Jacobian of the patch m dynamics evaluated at the equilibrium $\left[\tilde{R}_m^* = \frac{1-a+\sqrt{(1-a)^2+4kb}}{2b}, \tilde{I}_m^* = 0\right]$ is,

$$J\Big|_{\tilde{R}_m^*>0, \tilde{I}_m^*=0} = \begin{pmatrix} -\frac{r_R(b\tilde{R}_m^{*2}+k)}{k+\tilde{R}_m^*} & -r_R c_I \\ 0 & r_I(1-c_R\tilde{R}_m^*) \end{pmatrix}, \tag{S1.1}$$

which has eigenvalues $-\frac{r_R(b\tilde{R}_m^{*2}+k)}{k+\tilde{R}_m^*}$ and $r_I(1-c_R\tilde{R}_m^*)$. The equilibrium is stable if both of these eigenvalues are negative. The first eigenvalue is always negative and the second is negative for $c_R > \frac{2b}{1-a+\sqrt{(1-a)^2+4kb}}$.

The Jacobian matrix evaluated at the second equilibrium, $[\tilde{R}_m^* = 0, \tilde{I}_m^* = 1]$, is,

$$J\Big|_{\tilde{R}_m^*=0, \tilde{I}_m^*=1} = \begin{pmatrix} r_R\left(1-\frac{ac_I}{k}\right) & 0 \\ -r_I c_R & -r_I \end{pmatrix}. \tag{S1.2}$$

Thus, the equilibrium $[\tilde{R}_m^* = 0, \tilde{I}_m^* = 1]$ is stable whenever $c_I > \frac{k}{a}$. Because $\frac{k}{a} < 1$ (see main text), notice that any $c_I > 1$ will satisfy this condition.

We seek parameters that allow the microbe-independent plant to exclude the responsive plant in patch x ($[\tilde{R}_x^* = 0, \tilde{I}_x^* = 1]$ stable: $c_R > 1$) *and* the responsive plant to exclude the independent plant in patch m $\left(\left[\tilde{R}_m^* = \frac{1-a+\sqrt{(1-a)^2+4kb}}{2b}, \tilde{I}_m^* = 0\right] \text{stable: } c_R > \frac{2b}{1-a+\sqrt{(1-a)^2+4kb}}\right)$. These conditions define our parameter range of interest (main text inequalities (6)).

Notice that within this range, we always have alternative stable states in patch m ($[\tilde{R}_m^* = 0, \tilde{I}_m^* = 1]$ is always stable since $c_I > 1$ guarantees $c_I > \frac{k}{a}$). We also may have alternative stable states in patch x, if $c_R > 1$. There is, however, a range $\left(\frac{2b}{1-a+\sqrt{(1-a)^2+4kb}} < c_R < 1\right)$ where exclusion of the responsive plant occurs from all initial conditions.

The $D_R, D_I = \infty$ case

To study the dynamics with infinitely high dispersal rates, we follow the approach of Hastings (1982) and define new new variables: $R_s = \tilde{R}_m + \tilde{R}_x$, $R_d = \tilde{R}_m - \tilde{R}_x$, $I_s = \tilde{I}_m + \tilde{I}_x$, and $I_d = \tilde{I}_m - \tilde{I}_x$. As D_R and $D_I \to \infty$, differences between patches vanish and R_d and $I_d \to 0$, respectively. Dynamics of the remaining variables, R_s and I_s, are given by,

$$\frac{dR_s}{dt} = \frac{d\tilde{R}_m}{dt} + \frac{d\tilde{R}_x}{dt} = r_R\tilde{R}_m\left(1 - \frac{(\tilde{R}_m + c_I\tilde{I}_m)(a+b\tilde{R}_m)}{k+\tilde{R}_m}\right) + r_R\tilde{R}_x(1-\tilde{R}_x - c_I\tilde{I}_x) \tag{S1.3a}$$

$$\frac{dI_s}{dt} = \frac{d\tilde{I}_m}{dt} + \frac{d\tilde{I}_x}{dt} = r_I\tilde{I}_m(1-\tilde{I}_m - c_R\tilde{R}_m) + r_I\tilde{I}_x(1-\tilde{I}_x - c_R\tilde{R}_x) \tag{S1.3b}$$

Densities of the responsive plants in both patches converge to $\frac{R_s}{2}$: $\tilde{R}_m = \frac{R_s+R_d}{2} = \frac{R_s}{2}$ and $\tilde{R}_x = \frac{R_s-R_d}{2} = \frac{R_s}{2}$. Hereafter, we refer to $\frac{R_s}{2}$ simply as R. Likewise, the microbe-independent plant converges to a density that we will call I, equal to $\frac{I_s}{2}$, in both patches. We can then rewrite equations (S1.3) to give the dynamics in each patch under infinite dispersal,

$$\frac{dR}{dt} = \frac{r_R}{2}R\left(2 - \frac{(R+c_I I)(a+bR)}{k+R} - R - c_I I\right) \tag{S1.4a}$$

$$\frac{dI}{dt} = r_I I(1 - I - c_R R) \ . \tag{S1.4b}$$

The Jacobian matrix for equations (S1.4) is,

$$J = \begin{pmatrix} \frac{r_R}{2}\left(1 + \frac{2bR^3+(bk+a+c_I bI)R^2 - c_I akI}{(k+R)^2} - R - c_I I\right) & -\frac{c_R c_I R}{2}\left(\frac{a+bR}{k+R}+1\right) \\ -r_I c_R I & r_I(1-2I-c_R R) \end{pmatrix} . \tag{S1.5}$$

At the trivial equilibrium, $[R^* = I^* = 0]$, the eigenvalues of (S1.5) are $\frac{r_R}{2}$ and r_I, and so this equilibrium is never stable. The equilibrium where the microbe-independent plant excludes the responsive plant in both patches, $[R^* = 0, I^* = 1]$, has eigenvalues $\frac{r_R}{2}\left(1 - \frac{c_I a}{k} - c_I\right)$ and $-r_I$. The former is negative as long as $c_I > \frac{k}{k+a}$, which is automatically satisfied in our parameter range of interest, where $c_I > 1$. Thus, the microbe-independent plant can always stably exclude the responsive plant.

Model (S1.4) potentially has 2 additional types of equilibria: exclusion by the responsive plant and coexistence. Exclusion of the microbe-independent plant by the responsive plant occurs where $I^* = 0$ and R^* is a solution to,

$$(b+1)(R^*)^2 + (a+k-2)R^* - 2k = 0 \ . \tag{S1.6}$$

Because equation (S1.6) is concave up $(b+1>0)$ with a negative y-intercept $(-2k<0)$, it has only 1 positive root. This root is always real. Plugging the positive solutions to equation (S1.6) into the Jacobian (S1.5) yielded no stable solutions for over 1 million randomly selected parameter sets from our range of interest; we therefore believe that this equilibrium is never stable under infinite dispersal, although we have not proven it analytically.

Finally, there is a coexistence equilibrium where R^* is a solution to,

$$(b+1)(c_I c_R - 1)(R^*)^2 + ((c_I c_R - 1)(a+k) - c_I(b+1) + 2)R^* + 2k - c_I(a+k) = 0 \tag{S1.7}$$

and $I^* = 1 - c_R R^*$. From the same $> 10^6$ randomly drawn parameter sets mentioned above, none found a positive real root of (S1.7) that was $< \frac{1}{c_R}$ (which is needed for $I^* > 0$). Therefore, we conclude that coexistence is never feasible under infinite dispersal.

Realistic levels of plant dispersal ($0 < D_R, D_I < \infty$)

The Jacobian of the full 2-patch model (equations (5) in the main text) is

$$J\Big|_{\tilde{R}_m^*, \tilde{R}_x^*, \tilde{I}_m^*, \tilde{I}_x^*} = \tag{S1.8}$$

$$\left(\begin{array}{cc|cc} r_R(1-j(\tilde{R}_m^*,\tilde{I}_m^*))-D_R & D_R & -\frac{r_R c_I \tilde{R}_m^*(a+b\tilde{R}_m^*)}{k+\tilde{R}_m^*} & 0 \\ D_R & r_R(1-2\tilde{R}_x^*-c_I\tilde{I}_x^*)-D_R & 0 & -r_R c_R \tilde{R}_x^* \\ \hline -r_I c_R \tilde{I}_m^* & 0 & r_I(1-2\tilde{I}_m^*-c_R\tilde{R}_m^*)-D_I & D_I \\ 0 & -r_I c_R \tilde{I}_x^* & D_I & r_I(1-2\tilde{I}_x^*-c_R\tilde{R}_x^*)-D_I \end{array}\right),$$

with $j(\tilde{R}_m^*,\tilde{I}_m^*) = \frac{1}{(k+\tilde{R}_m^*)^2}\left[k(\tilde{R}_m^*+c_I\tilde{I}_m^*)(a+b\tilde{R}_m^*)+\tilde{R}_m^*(k+\tilde{R}_m^*)(a+2b\tilde{R}_m^*+bc_I\tilde{I}_m^*)\right]$.
The dashed lines in the Jacobian matrix (S1.8) divide the matrix into four 2×2 blocks, which is convenient for analysis because when the Jacobian is evaluated at several of the equilibria, the off-diagonal blocks (upper-right and/or lower-left) contain only zeros. In those cases, the eigenvalues of the entire Jacobian are the combined set of eigenvalues for the diagonal (upper-left and lower-right) blocks. Taking advantage of this and considering the diagonal blocks one at a time, rather than working with the entire Jacobian, is convenient because the Routh-Hurwitz criteria for eigenvalues < 0 (indicating a stable equilibrium point) are particularly simple when applied to 2×2 matrices.

Our model has four equilibria:

1. The trivial equilibrium: $\tilde{R}_m^* = \tilde{R}_x^* = \tilde{I}_m^* = \tilde{I}_x^* = 0$
2. Exclusion of the microbe-responsive plant in both patches: $\tilde{R}_m^* = \tilde{R}_x^* = 0$, $\tilde{I}_m^* = \tilde{I}_x^* = 1$
3. Exclusion of the microbe-resistant plant in both patches: $\tilde{R}_m^*, \tilde{R}_x^* > 0$, $\tilde{I}_m^* = \tilde{I}_x^* = 0$
4. Coexistence in both patches: $\tilde{R}_m^*, \tilde{R}_x^*, \tilde{I}_m^*, \tilde{I}_x^* > 0$

At the trivial equilibrium, both off-diagonal blocks of the Jacobian matrix contain only zeros so the eigenvalues of the entire Jacobian are the eigenvalues of the two diagonal blocks. These blocks both have the form,

$$\begin{pmatrix} r_s - D_s & D_s \\ D_s & r_s - D_s \end{pmatrix}, \tag{S1.9}$$

where $s = R$ in the upper-left block and I in the lower-right, and have the eigenvalues r_s and $r_s - 2D_s$. The eigenvalues of the Jacobian thus include both r_R and r_I, which are necessarily positive given that our parameters all have positive values. Therefore, the trivial equilibrium is always unstable.

When the microbe-responsive plant is excluded in both patches, the upper right block contains only zeros and again we can get all the eigenvalues from the diagonal blocks,

$$\begin{pmatrix} r_R(1-\frac{c_I a}{k}) - D_R & D_R \\ D_R & r_R(1-c_I) - D_R \end{pmatrix} \text{ and } \begin{pmatrix} -r_I - D_I & D_I \\ D_I & -r_I - D_I \end{pmatrix}. \tag{S1.10}$$

The second has eigenvalues $-r_I$ and $-r_I - 2D_I$, which are always negative. The eigenvalues of the first block are more complicated expressions, but we can apply the Routh-Hurwitz criteria to show that both are always negative as well. The Routh-Hurwitz

criteria guarantee that the eigenvalues of a 2×2 matrix are negative if the determinant of the matrix is positive and the trace is negative. Here, the determinant is,

$$\det\begin{pmatrix} r_R(1-\frac{c_I a}{k})-D_R & D_R \\ D_R & r_R(1-c_I)-D_R \end{pmatrix} = r_R^2(1-c_I)\left(1-\frac{c_I a}{k}\right)-D_R\left(2-c_I\left(1+\frac{a}{k}\right)\right). \quad \text{(S1.11)}$$

As explained in the main text, the definitions of our rescaled parameters necessarily makes $\frac{a}{k} > 1$. In addition, under the conditions for microbe-mediated competition, $c_I > 1$. Thus, $(1-c_I)$, $\left(1-\frac{c_I a}{k}\right)$, and $\left(2-c_I\left(1+\frac{a}{k}\right)\right)$ are all negative. The first term of (S1.11) is then positive and when we subtract the negative second term, the result will be positive and the first Routh-Hurwitz stability condition is satisfied. The trace of this block is,

$$\text{trace}\begin{pmatrix} r_R(1-\frac{c_I a}{k})-D_R & D_R \\ D_R & r_R(1-c_I)-D_R \end{pmatrix} = r_R\left(2-c_I\left(1+\frac{a}{k}\right)\right)-2D_R\ , \quad \text{(S1.12)}$$

which is clearly always negative, satisfying the second stability condition. Therefore, the equilibrium wherein the microbe-responsive plant is excluded from both patches is always stable under the conditions of microbe-mediated competition.

At the equilibrium where the responsive plant excludes the microbe-independent plant in both patches, $\tilde{I}_m^* = \tilde{I}_x^* = 0$ and $\tilde{R}_m^*$ is given by the implicit equation,

$$\sum_{q=0}^{5}\theta_q(\tilde{R}_m^*)^q = 0\ , \quad \text{(S1.13)}$$

with $\theta_5 = b^2\left(\frac{r_R}{D_R}\right)^3$, $\theta_4 = 2b\left(\frac{r_R}{D_R}\right)^2\left(\frac{r_R}{D_R}(a-1)+1\right)$,
$\theta_3 = \frac{r_R}{D_R}\left(\left(\frac{r_R}{D_R}(a-1)+1\right)^2 + b\left(1-\frac{r_R}{D_R}\right)\left(2\frac{r_R}{D_R}k+1\right)\right)$,
$\theta_2 = k\frac{r_R}{D_R}\left(1-\frac{r_R}{D_R}\right)\left(b+2\left(\frac{r_R}{D_R}(a-1)+1\right)\right)-1$,
$\theta_1 = k\left(\left(1-\frac{r_R}{D_R}\right)\left(\frac{r_R}{D_R}(a-1)+1+\left(1-\frac{r_R}{D_R}\right)\left(\frac{r_R}{D_R}k+1\right)\right)-2\right)$, and $\theta_0 = k^2\frac{r_R}{D_R}\left(\frac{r_R}{D_R}-2\right)$.
Then,

$$\tilde{R}_x^* = \tilde{R}_m^*\left(1-\frac{r_R}{D_R}\left(1-\frac{\tilde{R}_m^*(a+b\tilde{R}_m^*)}{k+\tilde{R}_m^*}\right)\right)\ . \quad \text{(S1.14)}$$

At the fourth equilibrium, where both species persist in both patches, it is not possible to write even an implicit equation for any of the equilibrium densities, and so we found the population densities at this final equilibrium by simulation.

The fact that we cannot explicitly solve for $\tilde{R}_m^*, \tilde{R}_x^* > 0$ limits our ability to derive useful stability criteria for the remaining two equilibria. We found the stability regions for these equilibria by finding the equilibrium densities for a given parameter combination using equations (S1.13)-(S1.14) (when the responsive plant excludes the microbe-independent plant) or by simulation (for coexistence), then plugging those densities into the full Jacobian matrix (S1.8) and finding the eigenvalues in Matlab.

References

Hastings, A. (1982) Dynamics of a single species in a spatially varying environment: the stabilizing role of high dispersal rates. *J Math Biol*, **16**, 49-55.
