# Supplementary material for: Spatial Heterogeneity in Soil Microbes Alters Outcomes of Plant Competition
Source: PLoS One. 2015 May 6;10(5):e0125788. doi: 10.1371/journal.pone.0125788 (PMC4422530; doi:10.1371/journal.pone.0125788)
Supplement: S2 Appendix — (PDF) [file pone.0125788.s002.pdf]

S2 Appendix: Effects of each parameter on stability of the equilibria

The figures in this appendix show the effects on the model's equilibrium behavior of changing each parameter. For comparison, dashed lines show the stability boundaries for the parameter values use in Fig. 2 in the main text ($r_R = 1.5$, $r_I = 1.5$, $c_R = 0.65$, $c_I = 1.1$, $a = 1.2$, $b = 0.01$, and $k = 1$). Shaded regions indicate stability of the equilibria when one of these parameters is changed. The equilibrium at which the microbe-independent plant excludes the microbe-responsive plant in both patches is always stable; white regions indicate where this is the only stable equilibrium present. In the dark gray region, the equilibrium at which responsive plant excludes the microbe-independent plant is stable and in the light gray region, the two species stably coexist.

(a) Higher r_R (b) Lower r_R

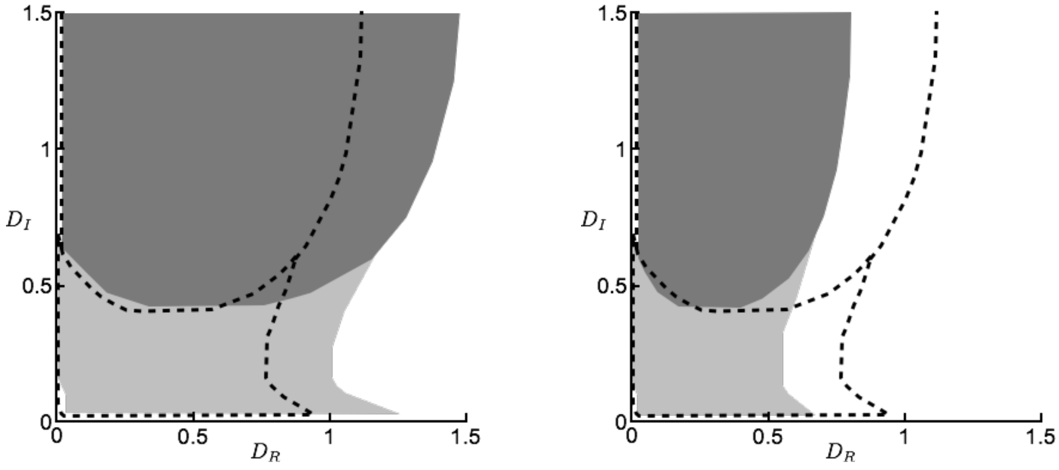

Figure S2.1. Effects of changing the parameter r_R ($r_R = 1.5$ is shown in Fig. 2 in the main text). (a) $r_R = 2$ and (b) $r_R = 1.1$.

(a) Higher r_I (b) Lower r_I

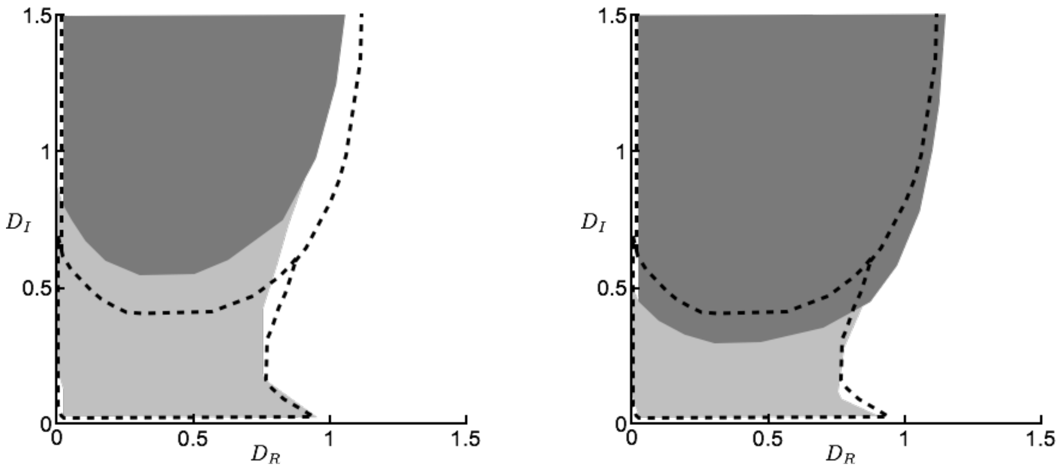

Figure S2.2. Effects of changing the parameter r_I ($r_I = 1.5$ is shown in Fig. 2 in the main text). (a) $r_I = 2$ and (b) $r_I = 1.1$.

(a) Higher c_R (b) Lower c_R

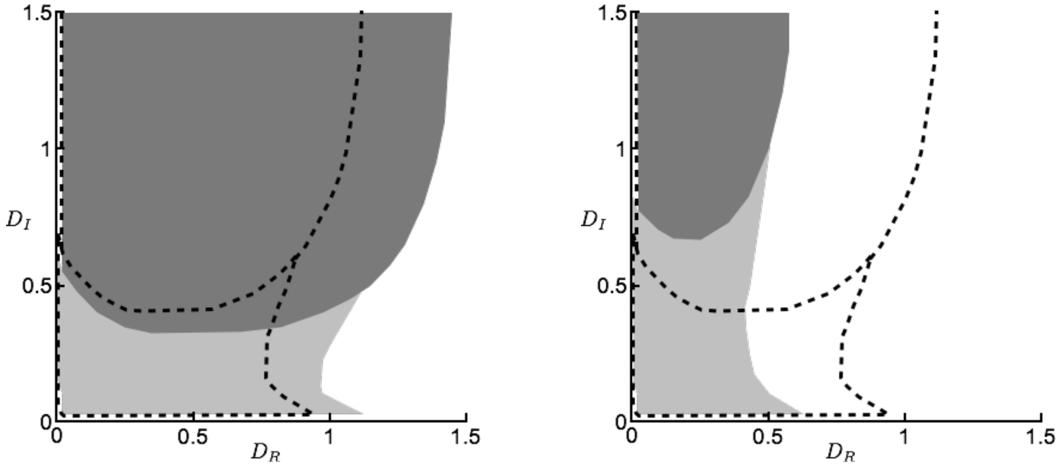

Figure S2.3. Effects of changing the parameter c_R ($c_R = 0.65$ is shown in Fig. 2 in the main text). (a) $c_R = 0.67$ and (b) $c_R = 0.6$.

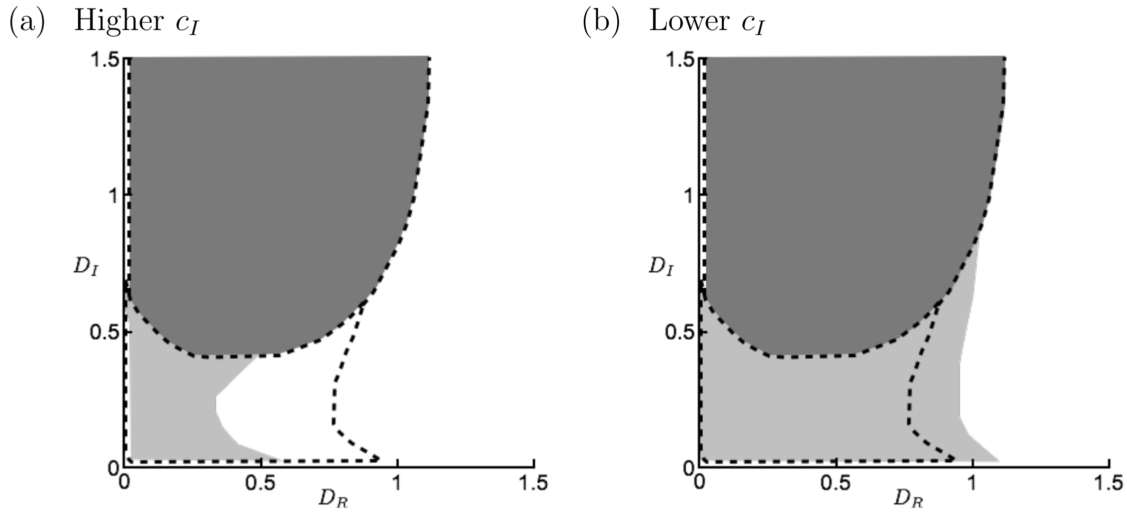

Figure S2.4. Effects of changing the parameter c_I ($c_I = 1.1$ is shown in Fig. 2 in the main text). (a) $c_I = 1.4$ and (b) $c_I = 1.01$.

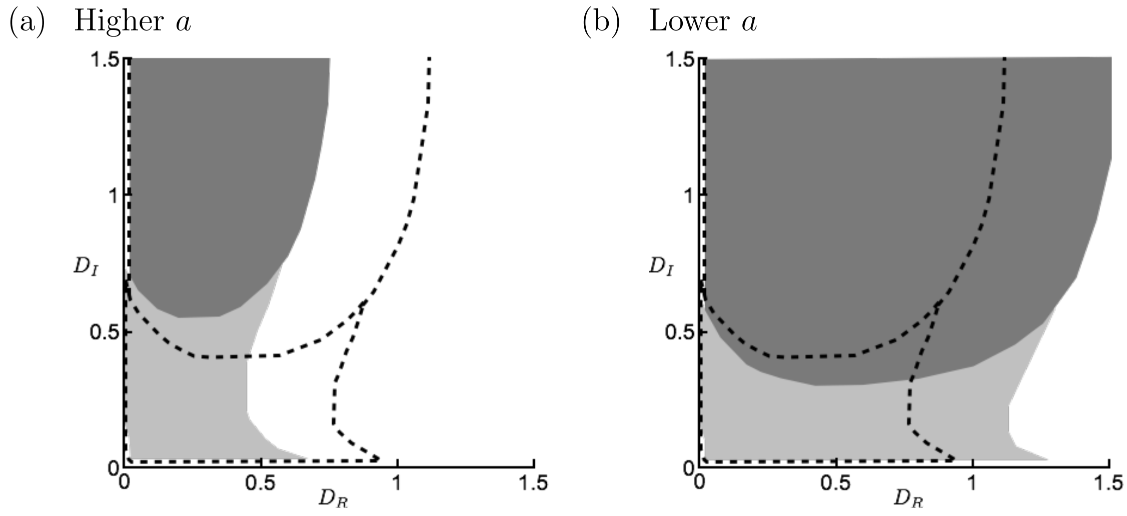

Figure S2.5. Effects of changing the parameter a ($a = 1.2$ is shown in Fig. 2 in the main text). (a) $a = 1.25$ and (b) $a = 1.15$.

(a) Higher b (b) Lower b

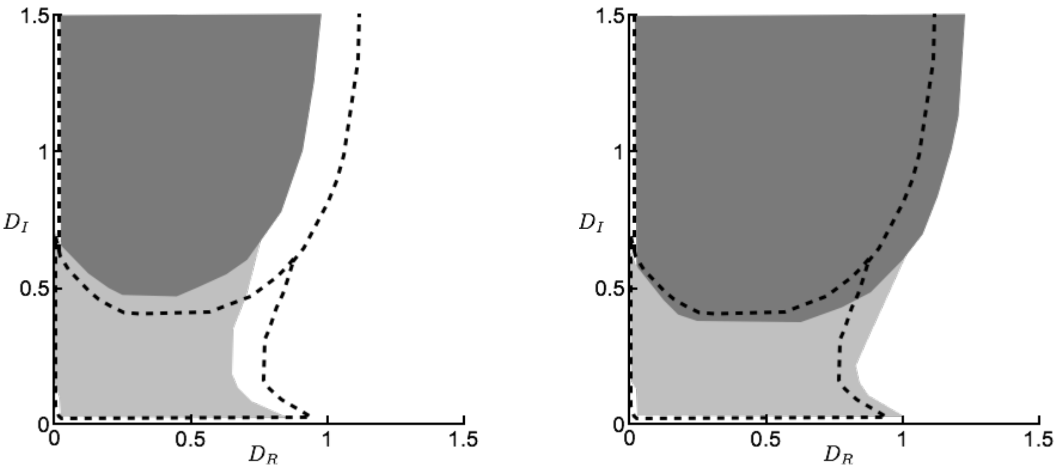

Figure S2.6. Effects of changing the parameter b ($b = 0.01$ is shown in Fig. 2 in the main text). (a) $b = 0.02$ and (b) $b = 0.001$.

(a) Higher k (b) Lower k

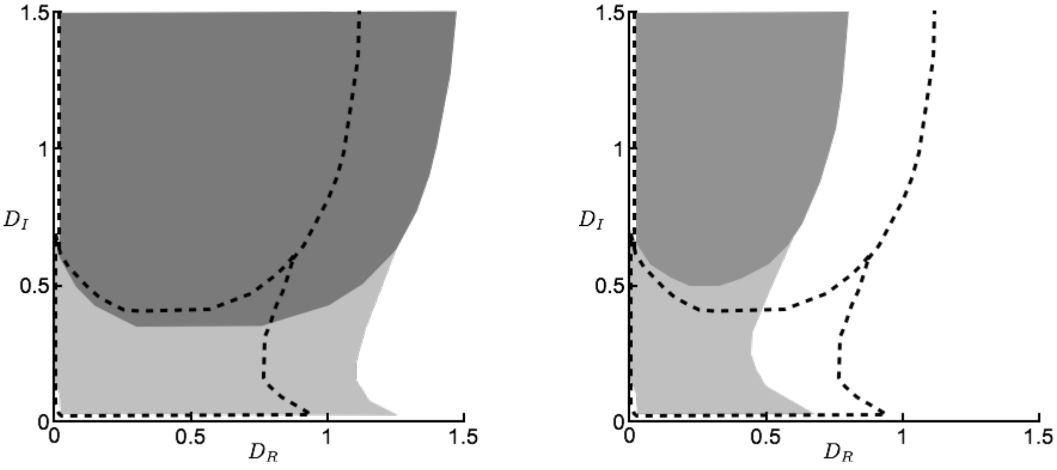

Figure S2.7. Effects of changing the parameter k ($k = 1$ is shown in Fig. 2 in the main text). (a) $k = 1.1$ and (b) $k = 0.9$.
